# Supplementary material for: Changes Induced by P2X7 Receptor Stimulation of Human Glioblastoma Stem Cells in the Proteome of Extracellular Vesicles Isolated from Their Secretome
Source: Cells. 2024 Mar 25;13(7):571. doi: 10.3390/cells13070571 (PMC11011151; doi:10.3390/cells13070571)
Supplement: Supplementary file 1 [file cells-13-00571-s001.zip › Scheme S1.pdf]

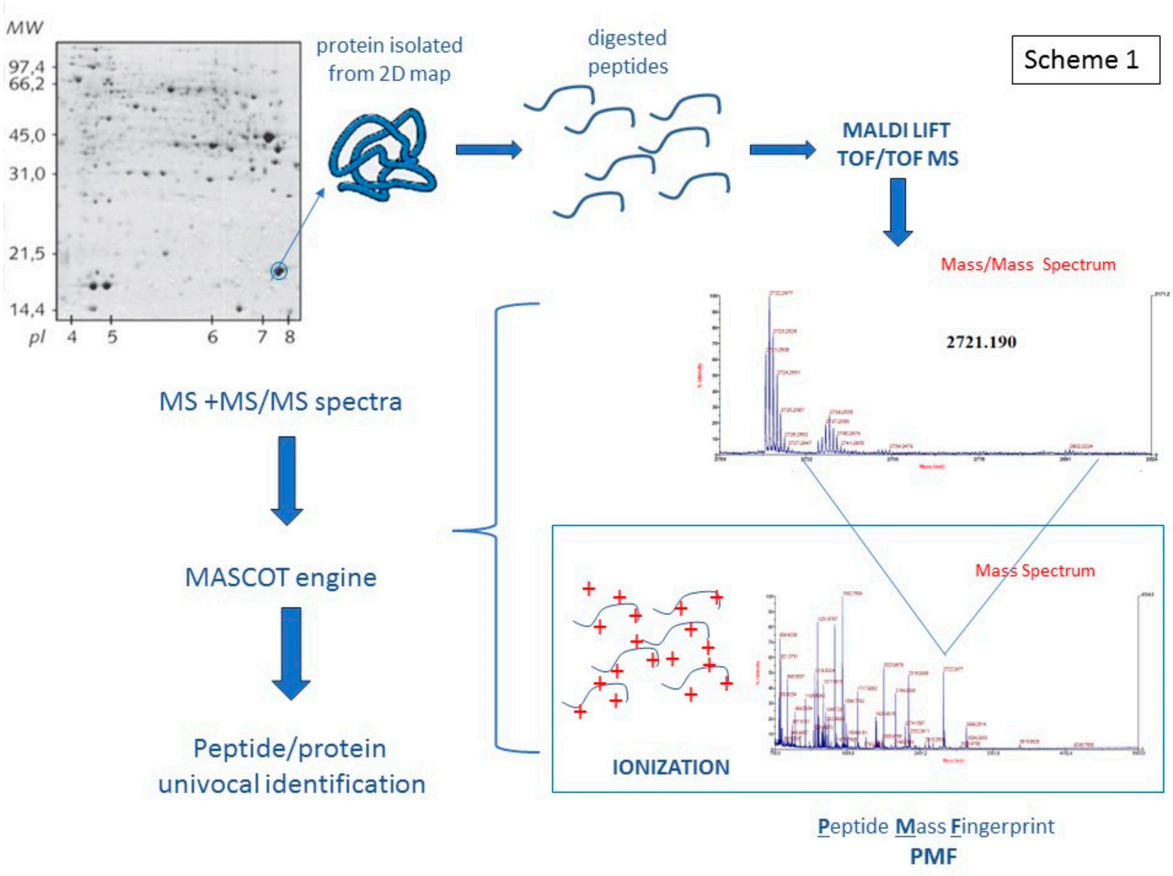

Proteins were separated by 2D electrophoresis and the picked protein spots were trypsinized into peptides; subsequently digested peptides underwent MALDI LIFT-TOF/TOF (Autoflex Speed, Bruker) analysis [Suckau D, Resemann A, Schuerenberg M, Hufnagel P, Franzen J, Holle A. A novel MALDI LIFT-TOF/TOF mass spectrometer for proteomics. Anal Bioanal Chem. 2003 Aug;376(7):952-65. doi: 10.1007/s00216-003-2057-0]. MS and MS/MS spectra obtained by this technique, integrated by BioTools 3.2 program, were launched into the Mascot database search engine generating a univocal protein sequence.
