# Supplementary material for: Changes Induced by P2X7 Receptor Stimulation of Human Glioblastoma Stem Cells in the Proteome of Extracellular Vesicles Isolated from Their Secretome
Source: Cells. 2024 Mar 25;13(7):571. doi: 10.3390/cells13070571 (PMC11011151; doi:10.3390/cells13070571)
Supplement: Supplementary file 1 [file cells-13-00571-s001.zip › Table S1.pdf]

Table S1. Principal characteristics of the proteins identified in GSC-derived MVs, the expression of which was modified by cell stimulation of P2X7R

a) Ex-novo induced proteins by P2X7R stimulation of GSCs			
Abbr. Name	Protein Name	Biological Process	Pathway
LMNB1	Laminin B1 (-)	Biological regulation and cell process: Nuclear migration and envelop organization; protein-containing complex localization	Fas signaling pathway
VIME	Vimentin (intermediate filament)	Cell process: Intermediate filament organization	None
ANXA5	Annexin A5 (calcium binding protein)	Signal transduction; negative regulation of apoptotic process *	Gonadotropin-releasing receptor pathway
DPYL2	Dihydropyrimidinase-related protein 2 (hydrolase)	Cell process, metabolic process: Pyrimidine nucleobase catabolic process	Axon guidance mediated by semaphoring; pyrimidine metabolism
CH60	60 kDa heat shock protein, mitochondrial (-)	Biological regulation and cell process, immune process, response to stimulus: Protein folding<br>Regulation of T cell activation	None
ATPB	ATP synthase subunit beta, mitochondrial (ATP synthase)	Cell process, metabolic process: ATP biosynthetic process; proton motive force-driven ATP synthesis; negative regulation of cell adhesion involved in substrate-bound cell migration *	ATP synthesis
ANXA2	Annexin A2 (calcium binding protein)	Cell process: Membrane raft assembly; collagen fibril organization	None
b) Changes in Top protein levels caused by P2X7R stimulation of GSCs			
Abbr. Name	Protein Name	Biological Process	Pathway
DPYL2	Dihydropyrimidinase-related protein 2 (hydrolase)	Cell process, metabolic process: Pyrimidine nucleobase catabolic process	None
HS90B	Heat shock protein HSP90-beta (Hsp90 family chaperone)	Biological regulation and cell process, response to stimulus: Protein folding and stabilization	None
QCR1	Cytochrome b-c1 complex subunit 1, mitochondrial (metalloprotease)	Cell process, metabolic process: Protein processing involved in protein targeting to mitochondrion	None
ACTB	Actin, cytoplasmic 1 (actin related protein)	Adherens junction assembly; cell motility; chromatin remodeling; positive regulation of cell differentiation; positive regulation of cell population proliferation *	Cadherin, Wnt, Integrin signaling; cytoskeletal regulation by Rho GTPase, Alzheimer (presenilin pathways) and Huntington diseases; inflammation mediated by chemokines and cytokines
CAZA1	F-actin-capping subunit alpha-1 (non-motor actin binding protein)	Biological regulation and cell process: Cytoskeleton organization	None
RUVB2	RuvB-like 2	Biological regulation and cell process, metabolic process: chromatin remodeling, protein-RNA complex assembly	None
CAPZB	F-actin-capping subunit beta (non-motor actin binding protein)	Biological regulation, cell process and developmental process: Cell morphogenesis	None
CPNE1	Copine-1 (calcium binding protein)	Biological regulation and cell process, response to stimulus: Response to oxygen-containing compound/organic substance; regulation of NF-kB and cytokine-mediated signaling	None

**PSME1**	**Proteasome activator complex subunit 1 (-)**	**Biological regulation: Regulation of proteasomal protein catabolic process; regulation of G1/S transition of mitotic cell cycle**	**None**
**PRDX4**	**Peroxiredoxin -4 (peroxidase)**	**Cell and homeostatic process, metabolic process, response to stimulus: Cell homeostasis, catabolic processes, response to oxidative stress**	**None**
**GRP75**	**Stress-70 protein (Hsp70 family chaperone)**	**inner mitochondrial membrane organization***	**Parkinson's disease**
**2AAA**	**Serine/Threonine-protein phoshatase 2, 65 kDa regulatory subunit A alpha (phosphatase modulator)**	**Cell process and metabolic process: Protein dephosphorylation; chromosome segregation**	**FGF signaling**
**TPIS**	**Triosephoshate isomerase (isomerase)**	**Cell process, metabolic process: Cell biosynthetic process, alcohol metabolic and glycolytic process; hexose biosynthetic and glucose metabolic process**	**Glycolysis**

* : No PANTHER category assigned; biological processes were found in UniProt database.
None: No pathway information available
