# Supplementary material for: Changes Induced by P2X7 Receptor Stimulation of Human Glioblastoma Stem Cells in the Proteome of Extracellular Vesicles Isolated from Their Secretome
Source: Cells. 2024 Mar 25;13(7):571. doi: 10.3390/cells13070571 (PMC11011151; doi:10.3390/cells13070571)
Supplement: Supplementary file 1 [file cells-13-00571-s001.zip › Table S2.pdf]

Table S2. Characterization of the proteins identified in both MV and EXOs derived from GSCs, the expression of which was modified by cell stimulation of P2X7R

Abbr. Name	Protein Description	Biological Process	Pathway
CSN4	COP9 signalosome complex subunit 4 (protease)	Protein deneddylation *	None
PRDX2	Peroxiredoxin-2 (peroxidase)	Cell homeostasis, response to oxidative/catabolic stress	None
PRDX4	Peroxiredoxin-4 (peroxidase)	Cell homeostasis, response to oxidative/catabolic stress	None
FRIL	Ferritin light chain (storage protein)	Intracellular iron ion homeostasis	PD
PSA6	Proteasome subunit alpha type-6 (protease)	Proteasomal catabolic process	None
ATP23	Mitochondrial inner membrane protease ATP homolog (metalloprotease)	Mitochondrial protein processing, mitochondrial proton-transporting ATP synthase complex assembly	None
ARP3	Actin-related protein 3 (actin and related protein)	Cell process: Arp2/3 complex-mediated actin nucleation	None
CLC1	Chloride intracellular channel protein 1 (ion channel)	Chloride transport	None

* : No PANTHER category assigned; biological processes were found in UniProt database.
None: No pathway information available
