# Supplementary material for: Changes Induced by P2X7 Receptor Stimulation of Human Glioblastoma Stem Cells in the Proteome of Extracellular Vesicles Isolated from Their Secretome
Source: Cells. 2024 Mar 25;13(7):571. doi: 10.3390/cells13070571 (PMC11011151; doi:10.3390/cells13070571)
Supplement: Supplementary file 1 [file cells-13-00571-s001.zip › Table S3.pdf]

Table S3. Characterization of the proteins identified in GSC-derived EXOs, the expression of which was modified by cell stimulation of P2X7R

a) Ex-novo induced proteins by P2X7R stimulation of GSCs			
Abbr. Name	Protein Description	Biological Process	Pathway
VIME	Vimentin (intermediate filament)	Cell process: intermediate filament organization	None
PSB9	Proteasome subunit beta type-9 (protease)	Metabolic process: proteasomal protein catabolic process	None
PSA6	Proteasome subunit alpha type-6 (protease)	Metabolic process: proteasomal protein catabolic process	Parkinson's disease (20S proteasome)
PSA5	Proteasome subunit alpha type-5 (protease)	Metabolic process: proteasomal protein catabolic process	Parkinson's disease (20S proteasome)
PSA2	Proteasome subunit alpha type-2 (protease)	Metabolic process: proteasomal protein catabolic process	Parkinson's disease (20S proteasome)
EF1G	Elongation factor 1-gamma	Cell process, metabolic process: translational elongation	None
RBBP4	Histone binding protein RBBP4	Biological regulator and cell process, metabolic process: chromatin remodeling, regulation of DNA-template transcription	None
b) Changes in Top protein levels caused by P2X7R stimulation of GSCs			
Abbr. Name	Protein Description	Biological Process	Pathway
TIMP2	Metalloproteinase inhibitor 2 (protease inhibitor)	Response to stimulus: Biological regulator	None
MMP2	72KDa type IV collagenase (metalloprotease)	Cell process and metabolic process, response to stimulus: Tissue remodeling, extracellular matrix organization, catabolic process	AD- Presenilin Pathway
MPP2+ GPSM2	MAGUK p55 sub-family member 2; G -protein signaling modulator 2	Cell process: Establishment of mitotic spindle orientation	Activator of G protein signaling
PDIA3	Protein disulfide-isomerase A3 (chaperone)	Cell process, response to stimulus: Protein folding, response to ER stress	None
IF4A1	Eukaryotic initiation factor 4 (RNA helicase)	Cell process, metabolic process: Cytoplasmic translational initiation	None
ATPB	ATP synthase subunit beta, Mitochondrial (ATP synthase)	Cell process, metabolic process: ATP biosynthetic process; proton motive force-driven ATP synthesis; negative regulation of cell adhesion involved in substrate-bound cell migration *	ATP synthesis
ENOG	Gamma enolase (lyase)	Metabolic process and Cell process: Glycolytic process	Glycolysis
ARP3	Actin-related protein 3	Cell process: Arp2/3 complex-mediated actin nucleation	None
SPB6	Serpin B6 (protease inhibitor)	negative regulation of endopeptidase activity*	None
ACTB	Actin, cytoplasmic 1 (actin related protein)	Adherens junction assembly; cell motility; chromatin remodeling; positive regulation of cell differentiation; positive regulation of cell population proliferation *	Cadherin, Wnt, Integrin signaling; cytoskeletal regulation by Rho GTPase, Alzheimer (presenilin pathways) and Huntington diseases; inflammation mediated by chemokines and cytokines

**TPM4**	**Tropomyosin alpha-4 chain (actin binding motor protein)**	**Cell process: Actin filament organization**	**None**
**POC1**	**Procollagen C-endopeptidase enhancer 1 (serine protease)**	**Metabolic process: Proteolysis**	**None**
**TIMP2**	**Metalloproteinase inhibitor 2 (protease inhibitor)**	**Metabolic process: Negative regulation of protein catabolic process and endopeptidase activity**	**None**
**CBPE**	**Carboxypeptidase E (protease)**	**Metabolic process: Peptide metabolic process**	**Vasopressin synthesis, CCKR signaling**
**ACTG**	**Actin, cytoplasmic 2 (actin related protein)**	**positive regulation of cell migration ***	**The same of actin B**

* : No PANTHER category assigned; biological processes were found in UniProt database.
None: No pathway information available
