# Supplementary material for: Changes Induced by P2X7 Receptor Stimulation of Human Glioblastoma Stem Cells in the Proteome of Extracellular Vesicles Isolated from Their Secretome
Source: Cells. 2024 Mar 25;13(7):571. doi: 10.3390/cells13070571 (PMC11011151; doi:10.3390/cells13070571)
Supplement: Supplementary file 1 [file cells-13-00571-s001.zip › Table S4.pdf]

Table S4. Usual role of the MV proteins codified by the genes reported in this table and upregulated in MVs from GSCs exposed to P2X7R stimulation. They likely interact each other and with the other proteins indicated below as predicted functional partners.

PPP2R1A	*Serine/threonine-protein phosphatase 2A 65 kDa regulatory subunit A alpha isoform; The PR65 subunit of protein phosphatase 2A serves as a scaffolding molecule to coordinate the assembly of the catalytic subunit and a variable regulatory B subunit. Upon interaction with GNA12 promotes dephosphorylation of microtubule associated protein TAU/MAPT. Required for proper chromosome segregation and for centromeric localization of SGO1 in mitosis. (589 aa)*
RUVBL2	*RuvB-like 2; Possesses single-stranded DNA-stimulated ATPase and ATP- dependent DNA helicase (5' to 3') activity; hexamerization is thought to be critical for ATP hydrolysis and adjacent subunits in the ring- like structure contribute to the ATPase activity. Component of the NuA4 histone acetyltransferase complex which is involved in transcriptional activation of select genes principally by acetylation of nucleosomal histones H4 and H2A. This modification may both alter nucleosome -DNA interactions and promote interaction of the modified histones with other proteins which positively re [...] (463 aa)*
ANXA5	*Annexin A5; This protein is an anticoagulant protein that acts as an indirect inhibitor of the thromboplastin-specific complex, which is involved in the blood coagulation cascade. (320 aa)*
HSPA9	*Stress-70 protein, mitochondrial; Chaperone protein which plays an important role in mitochondrial iron-sulfur cluster (ISC) biogenesis. Interacts with and stabilizes ISC cluster assembly proteins FXN, NFU1, NFS1 and ISCU. Regulates erythropoiesis via stabilization of ISC assembly. May play a role in the control of cell proliferation and cellular aging (By similarity). Belongs to the heat shock protein 70 family. (679 aa)*
PRDX4	*Peroxiredoxin-4; Thiol-specific peroxidase that catalyzes the reduction of hydrogen peroxide and organic hydroperoxides to water and alcohols, respectively. Plays a role in cell protection against oxidative stress by detoxifying peroxides and as sensor of hydrogen peroxide-mediated signaling events. Regulates the activation of NF-kappa-B in the cytosol by a modulation of I-kappa-B-alpha phosphorylation. (271 aa)*
UQCRC1	*Cytochrome b-c1 complex subunit 1, mitochondrial; Component of the ubiquinol-cytochrome c oxidoreductase, a multisubunit transmembrane complex that is part of the mitochondrial electron transport chain which drives oxidative phosphorylation. The respiratory chain contains 3 multisubunit complexes succinate dehydrogenase (complex II, CII), ubiquinol-cytochrome c oxidoreductase (cytochrome b-c1 complex, complex III, CIII) and cytochrome c oxidase (complex IV, CIV), that cooperate to transfer electrons derived from NADH and succinate to molecular oxygen, creating an electrochemical gradie [...] (480 aa)*
PSME1	*Proteasome activator complex subunit 1; Implicated in immunoproteasome assembly and required for efficient antigen processing. The PA28 activator complex enhances the generation of class I binding peptides by altering the cleavage pattern of the proteasome. (250 aa)*
ATP5F1B	*ATP synthase subunit beta, mitochondrial; Mitochondrial membrane ATP synthase (F(1)F(0) ATP synthase or Complex V) produces ATP from ADP in the presence of a proton gradient across the membrane which is generated by electron transport complexes of the respiratory chain. F-type ATPases consist of two structural domains, F(1) - containing the extramembraneous catalytic core, and F(0) - containing the membrane proton channel, linked together by a central stalk and a peripheral stalk. During catalysis, ATP synthesis in the catalytic domain of F(1) is coupled via a rotary mechanism of the c [...] (529 aa)*
HSP90AB1	*Heat shock protein HSP 90-beta; Molecular chaperone that promotes the maturation, structural maintenance and proper regulation of specific target proteins involved for instance in cell cycle control and signal transduction. Undergoes a functional cycle that is linked to its ATPase activity. This cycle probably induces conformational changes in the client proteins, thereby causing their activation. Interacts dynamically with various co-chaperones that modulate its substrate recognition, ATPase cycle and chaperone function. Engages with a range of client protein classes via its interact [...] (724 aa)*
HSPD1	*60 kDa heat shock protein, mitochondrial; Chaperonin implicated in mitochondrial protein import and macromolecular assembly. Together with Hsp10, facilitates the correct folding of imported proteins. May also prevent misfolding and promote the refolding and proper assembly of unfolded polypeptides generated under stress conditions in the mitochondrial matrix. The functional units of these chaperonins consist of heptameric rings of the large subunit Hsp60, which function as a back- to-back double ring. In a cyclic reaction, Hsp60 ring complexes bind one unfolded substrate protein per ri [...] (573 aa)*
CPNE1	*Copine-1; Calcium-dependent phospholipid-binding protein that plays a role in calcium-mediated intracellular processes. Involved in the TNF-alpha receptor signaling pathway in a calcium- dependent manner. Exhibits calcium-dependent phospholipid binding properties. Plays a role in neuronal progenitor cell differentiation; induces neurite outgrowth via a AKT-dependent signaling cascade and calcium- independent manner. May recruit target proteins to the cell membrane in a calcium-dependent manner. May function in membrane trafficking. Involved in TNF-alpha-induced NF-kappa-B transcription [...] (542 aa)*
DPYSL2	*Dihydropyrimidinase-related protein 2; Plays a role in neuronal development and polarity, as well as in axon growth and guidance, neuronal growth cone collapse and cell migration. Necessary for signaling by class 3 semaphorins and subsequent remodeling of the cytoskeleton. May play a role in endocytosis; Belongs to the metallo-dependent hydrolases superfamily. Hydantoinase/dihydropyrimidinase family. (677 aa)*
CAPZB	*F-actin-capping protein subunit beta; F-actin-capping proteins bind in a Ca(2+)-independent manner to the fast growing ends of actin filaments (barbed end) thereby blocking the exchange of subunits at these ends. Unlike other capping proteins (such as gelsolin and severin), these proteins do not sever actin filaments. Plays a role in the regulation of cell morphology and cytoskeletal organization. (301 aa)*
ACTB	*cell motility and contraction. In addition to their role in the cytoplasmic cytoskeleton, G- and F-actin also localize in the nucleus, and regulate gene transcription and motility and repair of damaged DNA. (375 aa)*
ANXA2	*Annexin A2; Calcium-regulated membrane-binding protein whose affinity for calcium is greatly enhanced by anionic phospholipids. It binds two calcium ions with high affinity. May be involved in heat-stress response. Inhibits PCSK9-enhanced LDLR degradation, probably reduces PCSK9 protein levels via a translational mechanism but also competes with LDLR for binding with PCSK9 ; Belongs to the annexin family. (357 aa)*

LMNB1	*Lamin-B1; Lamins are components of the nuclear lamina, a fibrous layer on the nucleoplasmic side of the inner nuclear membrane, which is thought to provide a framework for the nuclear envelope and may also interact with chromatin. (586 aa)*
CAPZA1	*F-actin-capping protein subunit alpha-1; F-actin-capping proteins bind in a Ca(2+)-independent manner to the fast growing ends of actin filaments (barbed end) thereby blocking the exchange of subunits at these ends. Unlike other capping proteins (such as gelsolin and severin), these proteins do not sever actin filaments. May play a role in the formation of epithelial cell junctions. (286 aa)*

Predicted Functional Partners:

		Score
FKBP4	*Peptidyl-prolyl cis-trans isomerase FKBP4, N-terminally processed; Immunophilin protein with PPIase and co-chaperone activities. Component of stero...*	0.999
RPAP3	*RNA polymerase II-associated protein 3; Forms an interface between the RNA polymerase II enzyme and chaperone/scaffolding protein, suggesting th...*	0.999
PPP2R5B	*Serine/threonine-protein phosphatase 2A 56 kDa regulatory subunit beta isoform; As the regulatory component of the serine/threonine-protein phosph...*	0.999
AHSA1	*Activator of 90 kDa heat shock protein ATPase homolog 1; Acts as a co-chaperone of HSP90AA1. Activates the ATPase activity of HSP90AA1 leading ...*	0.999
PSME2	*Proteasome activator complex subunit 2; Implicated in immunoproteasome assembly and required for efficient antigen processing. The PA28 activato...*	0.999
STUB1	*E3 ubiquitin-protein ligase CHIP; E3 ubiquitin-protein ligase which targets misfolded chaperone substrates towards proteasomal degradation. Collabor...*	0.999
PPP2CB	*Serine/threonine-protein phosphatase 2A catalytic subunit beta isoform; PP2A can modulate the activity of phosphorylase B kinase casein kinase 2, m...*	0.999
CDC37	*Hsp90 co-chaperone Cdc37, N-terminally processed; Co-chaperone that binds to numerous kinases and promotes their interaction with the Hsp90 co...*	0.999
PFN1	*Profilin-1; Binds to actin and affects the structure of the cytoskeleton. At high concentrations, profilin prevents the polymerization of actin, whereas it e...*	0.999
INO80B	*INO80 complex subunit B; Induces growth and cell cycle arrests at the G1 phase of the cell cycle.*	0.999
HSPE1	*10 kDa heat shock protein, mitochondrial; Co-chaperonin implicated in mitochondrial protein import and macromolecular assembly. Together with Hsp...*	0.999
ACTR5	*Actin-related protein 5; Proposed core component of the chromatin remodeling INO80 complex which is involved in transcriptional regulation, DNA repl...*	0.999
DNAJB1	*DnaJ homolog subfamily B member 1; Interacts with HSP70 and can stimulate its ATPase activity. Stimulates the association between HSC70 and HIP...*	0.999
CDK4	*Cyclin-dependent kinase 4; Ser/Thr-kinase component of cyclin D-CDK4 (DC) complexes that phosphorylate and inhibit members of the retinoblastom...*	0.999
COX5B	*Cytochrome c oxidase subunit 5B, mitochondrial; Component of the cytochrome c oxidase, the last enzyme in the mitochondrial electron transport cha...*	0.999
PPP2R5A	*Serine/threonine-protein phosphatase 2A 56 kDa regulatory subunit alpha isoform; The B regulatory subunit might modulate substrate selectivity and ...*	0.999
PIH1D1	*PIH1 domain-containing protein 1; Involved in the assembly of C/D box small nucleolar ribonucleoprotein (snoRNP) particles. Recruits the SWI/SNF co...*	0.999
SRCAP	*Helicase SRCAP; Catalytic component of the SRCAP complex which mediates the ATP-dependent exchange of histone H2AZ/H2B dimers for nucleoso...*	0.999
STRN	*Striatin; Calmodulin-binding protein which may function as scaffolding or signaling protein and may play a role in dendritic Ca(2+) signaling; Belongs to...*	0.999
GRPEL1	*GrpE protein homolog 1, mitochondrial; Essential component of the PAM complex, a complex required for the translocation of transit peptide-containin...*	0.999

The Tables reported above derive from the data analysis using the software STRING by (http://string-db.org/) to statistically determine the functions and pathways more likely associated with the protein list. The role of each predicted functional partner is explained below.

FKBP4_Peptidyl-prolyl cis-trans isomerase FKBP4, N-terminally processed;
Immunophilin protein with PPIase and co-chaperone activities. Component of steroid receptors heterocomplexes through interaction with heat-shock protein 90 (HSP90). May play a role in the intracellular trafficking of heterooligomeric forms of steroid hormone receptors between cytoplasm and nuclear compartments. The isomerase activity controls neuronal growth cones via regulation of TRPC1 channel opening. Acts also as a regulator of microtubule dynamics by inhibiting MAPT/TAU ability to promote microtubule assembly.

RIPAP3_ RNA polymerase II-associated protein 3;
Forms an interface between the RNA polymerase II enzyme and chaperone/scaffolding protein, suggesting that it is required to connect RNA polymerase II to regulators of protein complex formation. Belongs to the RPAP3 family.

PPP2R5B_ Serine/threonine-protein phosphatase 2A 56 kDa regulatory subunit beta isoform;
As the regulatory component of the serine/threonine-protein phosphatase 2A (PP2A) holoenzyme, modulates substrate specificity, subcellular localization, and responsiveness to phosphorylation. The phosphorylated form mediates the interaction between PP2A and AKT1, leading to AKT1 dephosphorylation.

AHSA1_ Activator of 90 kDa heat shock protein ATPase homolog 1;
Acts as a co-chaperone of HSP90AA1. Activates the ATPase activity of HSP90AA1 leading to increase in its chaperone activity. Competes with the inhibitory co- chaperone FNIP1 for binding to HSP90AA1, thereby providing a reciprocal regulatory mechanism for chaperoning of client proteins. Competes with the inhibitory co-chaperone TSC1 for binding to HSP90AA1, thereby providing a reciprocal regulatory mechanism for chaperoning of client proteins.

PSME2_Proteasome activator complex subunit 2;
Implicated in immunoproteasome assembly and required for efficient antigen processing. The PA28 activator complex enhances the generation of class I binding peptides by altering the cleavage pattern of the proteasome.

STUB1_ E3 ubiquitin-protein ligase CHIP;
E3 ubiquitin-protein ligase which targets misfolded chaperone substrates towards proteasomal degradation. Collaborates with ATXN3 in the degradation of misfolded chaperone substrates: ATXN3 restricting the length of ubiquitin chain attached to STUB1/CHIP substrates and preventing further chain extension. Ubiquitinates NOS1 in concert with Hsp70 and Hsp40. Modulates the activity of several chaperone complexes, including Hsp70, Hsc70 and Hsp90. Mediates transfer of non-canonical short ubiquitin chains to HSPA8 that have no effect on HSPA8 degradation.

PPP2CB_ Serine/threonine-protein phosphatase 2A catalytic subunit beta isoform;
PP2A can modulate the activity of phosphorylase B kinase casein kinase 2, mitogen-stimulated S6 kinase, and MAP-2 kinase. Belongs to the PPP phosphatase family. PP-1 subfamily.

CDC37_ Hsp90 co-chaperone Cdc37, N-terminally processed;
Co-chaperone that binds to numerous kinases and promotes their interaction with the Hsp90 complex, resulting in stabilization and promotion of their activity. Inhibits HSP90AA1 ATPase activity.

PFN1_Profilin-1;
Binds to actin and affects the structure of the cytoskeleton. At high concentrations, profilin prevents the polymerization of actin, whereas it enhances it at low concentrations. By binding to PIP2, it inhibits the formation of IP3 and DG. Inhibits androgen receptor (AR) and HTT aggregation and binding of G-actin is essential for its inhibition of AR.

INO80B_ INO80 complex subunit B;
Induces growth and cell cycle arrests at the G1 phase of the cell cycle.

HSPE1_ 10 kDa heat shock protein, mitochondrial;
Co-chaperonin implicated in mitochondrial protein import and macromolecular assembly. Together with Hsp60, facilitates the correct folding of imported proteins. May also prevent misfolding and promote the refolding and proper assembly of unfolded polypeptides generated under stress conditions in the mitochondrial matrix.

ACTR5_ Actin-related protein 5;
Proposed core component of the chromatin remodeling INO80 complex which is involved in transcriptional regulation, DNA replication and probably DNA repair. Involved in DNA double-strand break repair and UV-damage excision repair. Belongs to the actin family. ARP5 subfamily.

DNAJB1_ DnaJ homolog subfamily B member 1;
Interacts with HSP70 and can stimulate its ATPase activity. Stimulates the association between HSC70 and HIP. Negatively regulates heat shock-induced HSF1 transcriptional activity during the attenuation and recovery phase period of the heat shock response. Stimulates ATP hydrolysis and the folding of unfolded proteins mediated by HSPA1A/B (in vitro).

CDK4_ Cyclin-dependent kinase 4;
Ser/Thr-kinase component of cyclin D-CDK4 (DC) complexes that phosphorylate and inhibit members of the retinoblastoma (RB) protein family including RB1 and regulate the cell-cycle during G(1)/S transition. Phosphorylation of RB1 allows dissociation of the transcription factor E2F from the RB/E2F complexes and the subsequent transcription of E2F target genes which are responsible for the progression through the G(1) phase. Hypophosphorylates RB1 in early G(1) phase. Cyclin D-CDK4 complexes are major integrators of various mitogenenic and antimitogenic signals.

COX5B_ Cytochrome c oxidase subunit 5B, mitochondrial;

Component of the cytochrome c oxidase, the last enzyme in the mitochondrial electron transport chain which drives oxidative phosphorylation. The respiratory chain contains 3 multisubunit complexes succinate dehydrogenase (complex II, CII), ubiquinol- cytochrome c oxidoreductase (cytochrome b-c1 complex, complex III, CIII) and cytochrome c oxidase (complex IV, CIV), that cooperate to transfer electrons derived from NADH and succinate to molecular oxygen, creating an electrochemical gradient over the inner membrane.

PPP2R5A_ Serine/threonine-protein phosphatase 2A 56 kDa regulatory subunit alpha isoform;
The B regulatory subunit might modulate substrate selectivity and catalytic activity, and also might direct the localization of the catalytic enzyme to a particular subcellular compartment.

PIH1D1_ PIH1 domain-containing protein 1;
Involved in the assembly of C/D box small nucleolar ribonucleoprotein (snoRNP) particles. Recruits the SWI/SNF complex to the core promoter of rRNA genes and enhances pre-rRNA transcription. Mediates interaction of TELO2 with the R2TP complex which is necessary for the stability of MTOR and SMG1. Positively regulates the assembly and activity of the mTORC1 complex.

SRCAP_ Helicase SRCAP;
Catalytic component of the SRCAP complex which mediates the ATP-dependent exchange of histone H2AZ/H2B dimers for nucleosomal H2A/H2B, leading to transcriptional regulation of selected genes by chromatin remodeling. Acts as a coactivator for CREB-mediated transcription, steroid receptor-mediated transcription, and Notch- mediated transcription. Belongs to the SNF2/RAD54 helicase family. SWR1 subfamily.

STRN_ Striatin;
Calmodulin-binding protein which may function as scaffolding or signaling protein and may play a role in dendritic Ca(2+) signaling; Belongs to the WD repeat striatin family.

GRPEL1_ GrpE protein homolog 1, mitochondrial;
Essential component of the PAM complex, a complex required for the translocation of transit peptide-containing proteins from the inner membrane into the mitochondrial matrix in an ATP-dependent manner (By similarity). Seems to control the nucleotide-dependent binding of mitochondrial HSP70 to substrate proteins. It belongs to the GrpE family.
