# Supplementary material for: Changes Induced by P2X7 Receptor Stimulation of Human Glioblastoma Stem Cells in the Proteome of Extracellular Vesicles Isolated from Their Secretome
Source: Cells. 2024 Mar 25;13(7):571. doi: 10.3390/cells13070571 (PMC11011151; doi:10.3390/cells13070571)
Supplement: Supplementary file 1 [file cells-13-00571-s001.zip › Table S5.pdf]

Table S5. Usual role of the proteins, which are codified by the genes reported in this table, with upregulated expression in EXOs from GSCs exposed to P2X7R stimulation. They likely interact each other and with the other proteins indicated below as predicted functional partners

MMP2	*72 kDa type IV collagenase; Ubiquitinous metalloproteinase that is involved in diverse functions such as remodeling of the vasculature, angiogenesis, tissue repair, tumor invasion, inflammation, and atherosclerotic plaque rupture. As well as degrading extracellular matrix proteins, can also act on several nonmatrix proteins such as big endothelial 1 and beta- type CGRP promoting vasoconstriction. Also cleaves KISS at a Gly-|-Leu bond. Appears to have a role in myocardial cell death pathways. Contributes to myocardial oxidative stress by regulating the activity of GSK3beta. Cleaves GSK3 [...] (660 aa)*
ACTR3	*Actin-related protein 3; ATP-binding component of the Arp2/3 complex, a multiprotein complex that mediates actin polymerization upon stimulation by nucleation-promoting factor (NPF). The Arp2/3 complex mediates the formation of branched actin networks in the cytoplasm, providing the force for cell motility. Seems to contact the pointed end of the daughter actin filament. In addition to its role in the cytoplasmic cytoskeleton, the Arp2/3 complex also promotes actin polymerization in the nucleus, thereby regulating gene transcription and repair of damaged DNA. The Arp2/3 complex promote [...] (418 aa)*
CPE	*Carboxypeptidase E; Sorting receptor that directs prohormones to the regulated secretory pathway. Acts also as a prohormone processing enzyme in neuro/endocrine cells, removing dibasic residues from the C-terminal end of peptide hormone precursors after initial endoprotease cleavage. Belongs to the peptidase M14 family. (476 aa)*
EIF4A1	*Eukaryotic initiation factor 4A-I; ATP-dependent RNA helicase which is a subunit of the eIF4F complex involved in cap recognition and is required for mRNA binding to ribosome. In the current model of translation initiation, eIF4A unwinds RNA secondary structures in the 5'-UTR of mRNAs which is necessary to allow efficient binding of the small ribosomal subunit, and subsequent scanning for the initiator codon. (406 aa)*
PSMB5	*Proteasome subunit beta type-5; Component of the 20S core proteasome complex involved in the proteolytic degradation of most intracellular proteins. This complex plays numerous essential roles within the cell by associating with different regulatory particles. Associated with two 19S regulatory particles, forms the 26S proteasome and thus participates in the ATP- dependent degradation of ubiquitinated proteins. The 26S proteasome plays a key role in the maintenance of protein homeostasis by removing misfolded or damaged proteins that could impair cellular functions, and by removing pro [...] (263 aa)*
ATP5F1B	*ATP synthase subunit beta, mitochondrial; Mitochondrial membrane ATP synthase (F(1)F(0) ATP synthase or Complex V) produces ATP from ADP in the presence of a proton gradient across the membrane which is generated by electron transport complexes of the respiratory chain. F-type ATPases consist of two structural domains, F(1) - containing the extramembraneous catalytic core, and F(0) - containing the membrane proton channel, linked together by a central stalk and a peripheral stalk. During catalysis, ATP synthesis in the catalytic domain of F(1) is coupled via a rotary mechanism of the c [...] (529 aa)*
PSMA2	*Proteasome subunit alpha type-2; Component of the 20S core proteasome complex involved in the proteolytic degradation of most intracellular proteins. This complex plays numerous essential roles within the cell by associating with different regulatory particles. Associated with two 19S regulatory particles, forms the 26S proteasome and thus participates in the ATP- dependent degradation of ubiquitinated proteins. The 26S proteasome plays a key role in the maintenance of protein homeostasis by removing misfolded or damaged proteins that could impair cellular functions, and by removing pr [...] (234 aa)*
PDIA3	*Protein disulfide-isomerase A3; Protein disulfide isomerase family A member 3; Belongs to the protein disulfide isomerase family. (505 aa)*
PSMB9	*Proteasome subunit beta type-9; The proteasome is a multicatalytic proteinase complex which is characterized by its ability to cleave peptides with Arg, Phe, Tyr, Leu, and Glu adjacent to the leaving group at neutral or slightly basic pH. The proteasome has an ATP-dependent proteolytic activity. This subunit is involved in antigen processing to generate class I binding peptides. Replacement of PSMB6 by PSMB9 increases the capacity of the immunoproteasome to cleave model peptides after hydrophobic and basic residues. (219 aa)*
ACTB	*Actin, cytoplasmic 1, N-terminally processed; Actin is a highly conserved protein that polymerizes to produce filaments that form cross-linked networks in the cytoplasm of cells. Actin exists in both monomeric (G-actin) and polymeric (F-actin) forms, both forms playing key functions, such as cell motility and contraction. In addition to their role in the cytoplasmic cytoskeleton, G- and F-actin also localize in the nucleus, and regulate gene transcription and motility and repair of damaged DNA. (375 aa)*
ACTG1	*Actin, cytoplasmic 2, N-terminally processed; Actins are highly conserved proteins that are involved in various types of cell motility and are ubiquitously expressed in all eukaryotic cells. (375 aa)*
PSMB6	*Proteasome subunit beta type-6; Component of the 20S core proteasome complex involved in the proteolytic degradation of most intracellular proteins. This complex plays numerous essential roles within the cell by associating with different regulatory particles. Associated with two 19S regulatory particles, forms the 26S proteasome and thus participates in the ATP- dependent degradation of ubiquitinated proteins. The 26S proteasome plays a key role in the maintenance of protein homeostasis by removing misfolded or damaged proteins that could impair cellular functions, and by removing pro [...] (239 aa)*
SERPINB6	*Serpin B6; May be involved in the regulation of serine proteinases present in the brain or extravasated from the blood (By similarity). Inhibitor of cathepsin G, kallikrein-8 and thrombin. May play an important role in the inner ear in the protection against leakage of lysosomal content during stress and loss of this protection results in cell death and sensorineural hearing loss. (395 aa)*
ENO2	*Gamma-enolase; Has neurotrophic and neuroprotective properties on a broad spectrum of central nervous system (CNS) neurons. Binds, in a calcium- dependent manner, to cultured neocortical neurons and promotes cell survival (By similarity). (434 aa)*
EEF1G	*Elongation factor 1-gamma; Probably plays a role in anchoring the complex to other cellular components. (437 aa)*
TIMP2	*Metalloproteinase inhibitor 2; Complexes with metalloproteinases (such as collagenases) and irreversibly inactivates them by binding to their catalytic zinc cofactor. Known to act on MMP-1, MMP-2, MMP-3, MMP-7, MMP-8, MMP-9, MMP-10, MMP-13, MMP-14, MMP-15, MMP-16 and MMP-19. Belongs to the protease inhibitor I35 (TIMP) family. (220 aa)*

TPM4	*Tropomyosin alpha-4 chain; Binds to actin filaments in muscle and non-muscle cells. Plays a central role, in association with the troponin complex, in the calcium dependent regulation of vertebrate striated muscle contraction. Smooth muscle contraction is regulated by interaction with caldesmon. In non-muscle cells is implicated in stabilizing cytoskeleton actin filaments (By similarity). Binds calcium. (284 aa)*
GPSM2	*G-protein-signaling modulator 2; Plays an important role in mitotic spindle pole organization via its interaction with NUMA1. Required for cortical dynein-dynactin complex recruitment during metaphase. Plays a role in metaphase spindle orientation. Plays also an important role in asymmetric cell divisions. Has guanine nucleotide dissociation inhibitor (GDI) activity towards G(i) alpha proteins, such as GNAI1 and GNAI3, and thereby regulates their activity (By similarity). (684 aa)*
PCOLCE	*Procollagen C-endopeptidase enhancer 1; Binds to the C-terminal propeptide of type I procollagen and enhances procollagen C-proteinase activity. (449 aa)*

Predicted Functional Partners:

		Score
PSMA4	*Proteasome subunit alpha type-4; Component of the 20S core proteasome complex involved in the proteolytic degradation of most intracellular protei…*	0.999
PSMC4	*26S proteasome regulatory subunit 6B; Component of the 26S proteasome, a multiprotein complex involved in the ATP-dependent degradation of ubi…*	0.999
PSMD8	*26S proteasome non-ATPase regulatory subunit 8; Component of the 26S proteasome, a multiprotein complex involved in the ATP-dependent degrad…*	0.999
ATP5F1D	*ATP synthase subunit delta, mitochondrial; Mitochondrial membrane ATP synthase (F(1)F(0) ATP synthase or Complex V) produces ATP from ADP in …*	0.999
PSMA3	*Proteasome subunit alpha type-3; Component of the 20S core proteasome complex involved in the proteolytic degradation of most intracellular protei…*	0.999
PSMD7	*26S proteasome non-ATPase regulatory subunit 7; Component of the 26S proteasome, a multiprotein complex involved in the ATP-dependent degrad…*	0.999
WASL	*Neural Wiskott-Aldrich syndrome protein; Regulates actin polymerization by stimulating the actin- nucleating activity of the Arp2/3 complex. Involved i…*	0.999
PFN1	*Profilin-1; Binds to actin and affects the structure of the cytoskeleton. At high concentrations, profilin prevents the polymerization of actin, whereas it e…*	0.999
ARPC3	*Actin-related protein 2/3 complex subunit 3; Component of the Arp2/3 complex, a multiprotein complex that mediates actin polymerization upon stimul…*	0.999
ATP5F1E	*ATP synthase subunit epsilon, mitochondrial; Mitochondrial membrane ATP synthase (F(1)F(0) ATP synthase or Complex V) produces ATP from ADP …*	0.999
CANX	*Calnexin; Calcium-binding protein that interacts with newly synthesized glycoproteins in the endoplasmic reticulum. It may act in assisting protein ass…*	0.999
PSMB7	*Proteasome subunit beta type-7; Component of the 20S core proteasome complex involved in the proteolytic degradation of most intracellular protein…*	0.999
PSMC1	*26S proteasome regulatory subunit 4; Component of the 26S proteasome, a multiprotein complex involved in the ATP-dependent degradation of ubiq…*	0.999
PSMA6	*Proteasome subunit alpha type-6; Component of the 20S core proteasome complex involved in the proteolytic degradation of most intracellular protei…*	0.999
PSMD11	*26S proteasome non-ATPase regulatory subunit 11; Component of the 26S proteasome, a multiprotein complex involved in the ATP-dependent degra…*	0.999
PSMB1	*Proteasome subunit beta type-1; Component of the 20S core proteasome complex involved in the proteolytic degradation of most intracellular protein…*	0.999
ARPC1A	*Actin-related protein 2/3 complex subunit 1A; Probably functions as component of the Arp2/3 complex which is involved in regulation of actin polymeri…*	0.999
PSMD3	*26S proteasome non-ATPase regulatory subunit 3; Component of the 26S proteasome, a multiprotein complex involved in the ATP-dependent degrad…*	0.999
EIF4H	*Eukaryotic translation initiation factor 4H; Stimulates the RNA helicase activity of EIF4A in the translation initiation complex. Binds weakly mRNA.*	0.999
PSMA5	*Proteasome subunit alpha type-5; Component of the 20S core proteasome complex involved in the proteolytic degradation of most intracellular protei…*	0.999

The Tables reported above derive by the data analysis using the software STRING by (http://string-db.org/) to statistically determine the functions and pathways more likely associated with the protein list. The role of each predicted functional partner is explained below.

PSMA4_Proteasome subunit alpha type-4;

Component of the 20S core proteasome complex involved in the proteolytic degradation of most intracellular proteins. This complex plays numerous essential roles within the cell by associating with different regulatory particles. Associated with two 19S regulatory particles, forms the 26S proteasome and thus participates in the ATP- dependent degradation of ubiquitinated proteins. The 26S proteasome plays a key role in the maintenance of protein homeostasis by removing misfolded or damaged proteins that could impair cellular functions, regulates actin polymerization by stimulating the actin- nucleating activity of the Arp2/3 complex. Involved in various processes, such as mitosis and cytokinesis, via its role in the regulation of actin polymerization. Together with CDC42, involved in the extension and maintenance of the formation of thin, actin-rich surface projections called filopodia. In addition to its role in the cytoplasm, also plays a role in the nucleus by regulating gene transcription, probably by promoting nuclear actin polymerization.

PSMC4_26S proteasome regulatory subunit 6B;

Component of the 26S proteasome, a multiprotein complex involved in the ATP-dependent degradation of ubiquitinated proteins. This complex plays a key role in the maintenance of protein homeostasis by removing misfolded or damaged proteins, which could impair cellular functions, and by removing proteins whose functions are no longer required. Therefore, the proteasome participates in numerous cellular processes, including cell cycle progression, apoptosis, or DNA damage repair. PSMC4 belongs to the heterohexameric ring of AAA.

PSMD8_26S proteasome non-ATPase regulatory subunit 8;
Component of the 26S proteasome, a multiprotein complex involved in the ATP-dependent degradation of ubiquitinated proteins. This complex plays a key role in the maintenance of protein homeostasis by removing misfolded or damaged proteins, which could impair cellular functions, and by removing proteins whose functions are no longer required. Therefore, the proteasome participates in numerous cellular processes, including cell cycle progression, apoptosis, or DNA damage repair; Belongs to the proteasome subunit S14 family.

ATP5F1D_ ATP synthase subunit delta, mitochondrial;
Mitochondrial membrane ATP synthase (F(1)F(0) ATP synthase or Complex V) produces ATP from ADP in the presence of a proton gradient across the membrane which is generated by electron transport complexes of the respiratory chain. F-type ATPases consist of two structural domains, F(1) - containing the extramembraneous catalytic core, and F(0) - containing the membrane proton channel, linked together by a central stalk and a peripheral stalk.

PSMA3_ Proteasome subunit alpha type-3;
Component of the 20S core proteasome complex involved in the proteolytic degradation of most intracellular proteins. This complex plays numerous essential roles within the cell by associating with different regulatory particles. Associated with two 19S regulatory particles, forms the 26S proteasome and thus participates in the ATP- dependent degradation of ubiquitinated proteins. The 26S proteasome plays a key role in the maintenance of protein homeostasis by removing misfolded or damaged proteins that could impair cellular functions,Core component of nucleosome. Nucleosomes wrap and compact DNA into chromatin, limiting DNA accessibility to the cellular machineries which require DNA as a template. Histones thereby play a central role in transcription regulation, DNA repair, DNA replication and chromosomal stability. DNA accessibility is regulated via a complex set of post-translational modifications of histones, also called histone code, and nucleosome remodeling.

PSMD7_ 26S proteasome non-ATPase regulatory subunit 7;
Component of the 26S proteasome, a multiprotein complex involved in the ATP-dependent degradation of ubiquitinated proteins. This complex plays a key role in the maintenance of protein homeostasis by removing misfolded or damaged proteins, which could impair cellular functions, and by removing proteins whose functions are no longer required. Therefore, the proteasome participates in numerous cellular processes, including cell cycle progression, apoptosis, or DNA damage repair; Belongs to the peptidase M67A family._

WASL_ Neural Wiskott-Aldrich syndrome protein;
Regulates actin polymerization by stimulating the actin- nucleating activity of the Arp2/3 complex. Involved in various processes, such as mitosis and cytokinesis, via its role in the regulation of actin polymerization. Together with CDC42, involved in the extension and maintenance of the formation of thin, actin-rich surface projections called filopodia. In addition to its role in the cytoplasm, also plays a role in the nucleus by regulating gene transcription, probably by promoting nuclear actin polymerization.

PFN1_Profilin-1;
Binds to actin and affects the structure of the cytoskeleton. At high concentrations, profilin prevents the polymerization of actin, whereas it enhances it at low concentrations. By binding to PIP2, it inhibits the formation of IP3 and DG. Inhibits androgen receptor (AR) and HTT aggregation and binding of G-actin is essential for its inhibition of AR.

ARPC3_ Actin-related protein 2/3 complex subunit 3;
Component of the Arp2/3 complex, a multiprotein complex that mediates actin polymerization upon stimulation by nucleation-promoting factor (NPF). The Arp2/3 complex mediates the formation of branched actin networks in the cytoplasm, providing the force for cell motility. In addition to its role in the cytoplasmic cytoskeleton, the Arp2/3 complex also promotes actin polymerization in the nucleus, thereby regulating gene

transcription and repair of damaged DNA. The Arp2/3 complex promotes homologous recombination (HR) repair in response to DNA stress.

ATP5F1E_ATP synthase subunit epsilon, mitochondrial;
Mitochondrial membrane ATP synthase (F(1)F(0) ATP synthase or Complex V) produces ATP from ADP in the presence of a proton gradient across the membrane which is generated by electron transport complexes of the respiratory chain. F-type ATPases consist of two structural domains, F(1) - containing the extramembraneous catalytic core, and F(0) - containing the membrane proton channel, linked together by a central stalk and a peripheral stalk.

CANX_Calnexin;
Calcium-binding protein that interacts with newly synthesized glycoproteins in the endoplasmic reticulum. It may act in assisting protein assembly and/or in the retention within the ER of unassembled protein subunits. It seems to play a major role in the quality control apparatus of the ER by the retention of incorrectly folded proteins. Associated with partial T-cell antigen receptor complexes that escape the ER of immature thymocytes, it may function as a signaling complex regulating thymocyte maturation. Additionally it may play a role in receptor-mediated endocytosis.

PSMAB7_Proteasome subunit beta type-7;
Component of the 20S core proteasome complex involved in the proteolytic degradation of most intracellular proteins. This complex plays numerous essential roles within the cell by associating with different regulatory particles. Associated with two 19S regulatory particles, forms the 26S proteasome and thus participates in the ATP- dependent degradation of ubiquitinated proteins. The 26S proteasome plays a key role in the maintenance of protein homeostasis by removing misfolded or damaged proteins that could impair cellular functions,

PSMC1_ 26S proteasome regulatory subunit 4; Component of the 26S proteasome, a multiprotein complex involved in the ATP-dependent degradation of ubiquitinated proteins. This complex plays a key role in the maintenance of protein homeostasis by removing misfolded or damaged proteins, which could impair cellular functions, and by removing proteins whose functions are no longer required. Therefore, the proteasome participates in numerous cellular processes, including cell cycle progression, apoptosis, or DNA damage repair.

PSMA6_ Proteasome subunit alpha type-6;
Component of the 20S core proteasome complex involved in the proteolytic degradation of most intracellular proteins. This complex plays numerous essential roles within the cell by associating with different regulatory particles. Associated with two 19S regulatory particles, forms the 26S proteasome and thus participates in the ATP- dependent degradation of ubiquitinated proteins.

PSMD11_ 6S proteasome non-ATPase regulatory subunit 11;
Component of the 26S proteasome, a multiprotein complex involved in the ATP-dependent degradation of ubiquitinated proteins. This complex plays a key role in the maintenance of protein homeostasis by removing misfolded or damaged proteins, which could impair cellular functions, and by removing proteins whose functions are no longer required. Therefore, the proteasome participates in numerous cellular processes, including cell cycle progression, apoptosis, or DNA damage repair.

PSMB1_ Proteasome subunit beta type-1;
Component of the 20S core proteasome complex involved in the proteolytic degradation of most intracellular proteins. This complex plays numerous essential roles within the cell by associating with different regulatory particles. Associated with two 19S regulatory particles, forms the 26S proteasome and thus participates in the ATP- dependent degradation of ubiquitinated proteins. The 26S proteasome plays a key role in the

maintenance of protein homeostasis by removing misfolded or damaged proteins that could impair cellular functions

ARPC1_ Actin-related protein 2/3 complex subunit 1A;

Probably functions as component of the Arp2/3 complex which is involved in regulation of actin polymerization and together with an activating nucleation-promoting factor (NPF) mediates the formation of branched actin networks; Belongs to the WD repeat ARPC1 family.

PSMD3_ 26S proteasome non-ATPase regulatory subunit 3;

Component of the 26S proteasome, a multiprotein complex involved in the ATP-dependent degradation of ubiquitinated proteins. This complex plays a key role in the maintenance of protein homeostasis by removing misfolded or damaged proteins, which could impair cellular functions, and by removing proteins whose functions are no longer required. Therefore, the proteasome participates in numerous cellular processes, including cell cycle progression, apoptosis, or DNA damage repair; Belongs to the proteasome subunit S3 family.

EIF4H_ Eukaryotic translation initiation factor 4H;

Stimulates the RNA helicase activity of EIF4A in the translation initiation complex. Binds weakly mRNA.

PSMA5_ Proteasome subunit alpha type-5;

Component of the 20S core proteasome complex involved in the proteolytic degradation of most intracellular proteins. This complex plays numerous essential roles within the cell by associating with different regulatory particles. Associated with two 19S regulatory particles, forms the 26S proteasome and thus participates in the ATP- dependent degradation of ubiquitinated proteins. The 26S proteasome plays a key role in the maintenance of protein homeostasis by removing misfolded or damaged proteins that could impair cellular functions,
